# Association between the skeletal muscle-to-visceral fat area ratio and liver fibrosis risk in patients with non-alcoholic fatty liver disease: a cross-sectional study

**DOI:** 10.3389/fpubh.2026.1816286

**Published:** 2026-07-15

**Authors:** Jingru Liu, Xitong Jiao, Lu Liu, Anqi Chen, Qixue Kang, Jiayue Guo, Yuxing Wang, Yanmeng Qi, Lili You

**Affiliations:** 1 School of Health Policy and Management, Chinese Academy of Medical Sciences and Peking Union Medical College, Beijing, China; 2 International Medical Service of Peking Union Medical College Hospital (Xidan Branch), Beijing, China

**Keywords:** body composition (sarcopenic obesity), FIB-4 index, liver fibrosis, non-alcoholic fatty liver disease (NAFLD), skeletal muscle-to-visceral fat area ratio (SVR)

## Abstract

**Background:**

Non-alcoholic fatty liver disease (NAFLD) is highly prevalent and closely linked to metabolic dysfunction, with progressive liver fibrosis driving adverse outcomes. Conventional obesity metrics such as body mass index (BMI) and waist circumference inadequately capture body-composition imbalance. The skeletal muscle-to-visceral fat area ratio (SVR) integrates muscle reserve and visceral adiposity and may better reflect metabolic risk. However, large-scale evidence on SVR and fibrosis risk in Chinese patients with NAFLD is limited. This study investigated the association between the SVR and liver fibrosis risk in patients with non-alcoholic fatty liver disease (NAFLD).

**Methods:**

A cross-sectional study was conducted involving 661 patients with fatty liver from the Health Management Center of Peking Union Medical College Hospital (Xidan Branch) between January 2021 and November 2024. SVR was measured using a body composition analyzer, and the fibrosis-4 index (FIB-4) was calculated. Because the raw FIB-4 score was right-skewed and the residuals from the raw FIB-4 linear model were not normally distributed, log(FIB-4) was used as the continuous outcome. Hierarchical multivariable linear regression was used to analyze the association between SVR and log(FIB-4), and the mediating role of BMI was tested using the bootstrap method.

**Results:**

The raw FIB-4 score was markedly right-skewed (skewness = 1.440, Shapiro–Wilk *p* < 0.001), whereas the distribution of log(FIB-4) was substantially improved after natural logarithmic transformation (skewness = −0.123, Shapiro–Wilk *p* = 0.444). In the unadjusted model, SVR was inversely associated with log(FIB-4) (B = −0.067, 95% CI −0.109 to −0.025, *p* = 0.002). In the fully adjusted model including BMI, the inverse association between SVR and log(FIB-4) became stronger. Mediation analysis revealed a significant suppression effect of BMI, with a positive indirect effect and a negative direct effect (indirect effect = 0.041, direct effect = −0.108). Subgroup analysis showed that the suppression effect was observed in the higher-BMI subgroup and in both men and women, but not in the lower-BMI subgroup.

**Conclusion:**

Higher SVR was associated with lower log(FIB-4) among patients with NAFLD, and BMI appeared to suppress part of this association. These findings suggest that SVR may provide body-composition information beyond BMI in FIB-4-based risk assessment, but longitudinal studies with more precise fibrosis assessment are needed for validation.

## 1 Introduction

Non-alcoholic fatty liver disease (NAFLD) is the most prevalent chronic liver disease worldwide and is closely linked to metabolic disorders such as obesity, hypertension, hyperglycaemia, and dyslipidaemia (1). The clinical spectrum of NAFLD ranges from simple hepatic steatosis to non-alcoholic steatohepatitis (NASH), cirrhosis, and hepatocellular carcinoma (HCC) (2, 3), collectively imposing a substantial disease burden (4, 5). However, NAFLD is not merely a liver condition; rather, it represents the hepatic manifestation of the metabolic syndrome (6). Therefore, accurate assessment and early identification of metabolic risk factors that predispose individuals with NAFLD to progressive liver fibrosis are crucial for optimizing clinical management and improving patient outcomes (7).

At present, clinical practice commonly relies on body mass index (BMI) and waist circumference to assess obesity and related metabolic risk. However, BMI does not distinguish fat from lean mass or capture their distribution, and although waist circumference reflects central adiposity, it does not reliably differentiate subcutaneous from visceral fat (8). Alterations in body composition are increasingly recognized as key determinants of cardiovascular disease (CVD) risk (9). In this context, the skeletal muscle-to-visceral fat area ratio (SVR) simultaneously quantifies muscle reserve and visceral adipose burden. Several other body composition indices have been used to characterize sarcopenia, sarcopenic obesity, or fat-muscle imbalance (10). For example, SMI is commonly used to quantify relative skeletal muscle mass, and consensus-based sarcopenia definitions, including EWGSOP2 and AWGS 2019, incorporate low muscle mass together with muscle strength and physical performance (11, 12). Fat-to-muscle ratio and related composite indices further capture the imbalance between adiposity and skeletal muscle (13). However, these indicators either focus mainly on the muscle component or do not specifically distinguish visceral adiposity from other fat compartments. SVR was selected in this study because it integrates two metabolically relevant components, skeletal muscle mass and visceral fat area, into a single measure (14, 15). This feature is particularly relevant to NAFLD, in which both reduced muscle reserve and excess visceral adiposity may contribute to insulin resistance, systemic inflammation, and liver fibrosis progression (16–18). A lower SVR indicates excess visceral fat accompanied by insufficient skeletal muscle mass, a pattern of body-composition imbalance that may reflect a more severe metabolic dysregulation phenotype (19). A 4-year retrospective cohort study from Japan reported that, among 92 patients with NAFLD, declines in SVR during follow-up were associated with worsening hepatic steatosis and fibrosis (14). A separate 4-year retrospective cohort study from Korea further showed that, among individuals without NAFLD at baseline, lower SVR was associated with a higher incidence of NAFLD and an increased risk of liver fibrosis during follow-up (15). In addition, a 3-year retrospective cohort study from Korea found that patients with sarcopenic obesity (SO) had a higher proportion of incident liver fibrosis and cirrhosis over the follow-up period (20).

Liver biopsy remains the gold standard for the diagnosis of hepatic fibrosis (21). However, because it is invasive and associated with procedure-related risks and substantial costs, it is not suitable as a screening tool in large populations (22). Recent clinical guidelines indicate that the FIB-4 is a feasible, non-invasive approach for risk stratification and prediction of liver fibrosis (23–25). Although previous studies have separately examined the associations of sarcopenia or visceral adiposity with adverse outcomes in NAFLD, evidence from large-scale population-based studies that integrate both components into a single metric (SVR) and systematically evaluate its association with fibrosis risk in patients with fatty liver disease remains limited. This evidence gap is particularly pronounced in Chinese populations. To address this gap, we analyzed retrospective health examination data from the Health Management Center of Peking Union Medical College Hospital, Xidan Campus. Using FIB-4 as the outcome measure, we examined the association between SVR and FIB-4 among patients with NAFLD, with the aim of identifying informative check-up-based indicators to support risk assessment and ultimately improve quality of life in this population (26).

## 2 Materials and methods

### 2.1 Study design and participants

We identified 903 individuals with fatty liver disease who underwent health examinations at the Health Management Center of Peking Union Medical College Hospital, Xidan Campus, between January 2021 and November 2024. In this retrospective study, NAFLD was used as the operational diagnostic framework. Participant identification was based on ultrasonographic evidence of fatty liver disease recorded in the health examination system, together with the exclusion of excessive alcohol consumption and other major liver diseases according to the available clinical records (27). This approach was used to maintain consistency with the original case definition and comparability with previous studies on SVR, body composition, and FIB-4 conducted under the NAFLD framework.

#### 2.1.1 Inclusion criteria

Participants were eligible if they (1) were aged 18 years or older, (2) had complete laboratory records, and (3) had fatty liver disease diagnosed by abdominal ultrasonography.

#### 2.1.2 Exclusion criteria

We excluded individuals who (1) had used liver enzyme-lowering medications (including biphenyl dimethyl dicarboxylate, bicyclol, or Wuzhi capsule) within the past 3 months, (2) had documented or self-reported viral hepatitis, other infectious diseases, autoimmune diseases, malignancy, or severe hepatic or renal insufficiency, or (3) had excessive alcohol consumption, defined as self-reported alcohol intake exceeding the sex-specific thresholds of >140 g/week for women or >210 g/week for men. Alcohol consumption was assessed by self-report at enrollment. Participants with alcohol intake below these thresholds were not excluded on the basis of alcohol use alone. After applying these criteria, 661 participants were included in the final analysis, representing 73.2% of the initial sample.

#### 2.1.3 Ethics statement

The study was conducted in accordance with the Declaration of Helsinki and was approved by the Ethics Committee of Peking Union Medical College Hospital (approval number K7982). All participants provided informed consent.

### 2.2 Measurements

#### 2.2.1 Data collection

We collected seven domains of health examination data, including sex, age, medical history, smoking status, medication use, body composition measures, and laboratory parameters. Body composition measures included body mass index (BMI, kg/m^2^), percentage body fat (PBF, %), waist-to-hip ratio (WHR), appendicular skeletal muscle mass (ASM), skeletal muscle index (SMI), and the skeletal muscle-to-visceral fat area ratio (SVR). PBF was calculated as body fat mass (kg) divided by body weight (kg) multiplied by 100. WHR was calculated as waist circumference (WC) divided by hip circumference (HC). ASM was calculated as the sum of muscle mass in both arms and both legs. SMI was calculated as ASM divided by height squared (m^2^). Laboratory parameters included platelet count (PLT), alanine aminotransferase (ALT), albumin (ALB), aspartate aminotransferase (AST), total cholesterol (TC), triglycerides (TG), and high-density lipoprotein cholesterol (HDL-C), among others. We calculated the fibrosis-4 index (FIB-4) using the following formula: FIB-4 = age (years) × AST (U/L)/(PLT (×10^9^/L) × √ALT (U/L)). FIB-4 categories were defined in accordance with guideline-recommended thresholds (25, 27). A FIB-4 value below 1.3 was classified as low risk, with an estimated probability of progression to advanced fibrosis or cirrhosis of less than 3%. A FIB-4 value between 1.3 and 2.67 was classified as intermediate risk, for which further assessment using transient elastography or serum biomarkers was recommended (25). A FIB-4 value above 2.67 was classified as high risk and indicated a substantially increased risk of fibrosis progression.

#### 2.2.2 Measurement procedures

##### 2.2.2.1 Diagnosis of fatty liver disease

Fatty liver disease was assessed by abdominal ultrasonography using a PHILP-IU colour Doppler ultrasound system with a probe frequency of 2.8 to 5.0 MHz. All examinations were performed by qualified technical staff with an academic rank of associate professor or above. Fatty liver disease was diagnosed based on ultrasonographic findings that met one or more of the following criteria: (1) diffuse enhancement of near-field echoes in the hepatic region (greater than that of the kidney and spleen) with gradual attenuation of far-field echoes; (2) poor visualization of intrahepatic ductal structures; (3) mild to moderate hepatomegaly with rounded and blunted liver margins; (4) reduced or difficult-to-detect intrahepatic colour flow signals on color Doppler imaging with preserved normal vascular course; and (5) incomplete or unclear visualization of the capsule of the right hepatic lobe and the diaphragm. Participants with fatty liver disease on ultrasonography were included in the study.

##### 2.2.2.2 Body composition assessment

Body composition was measured using the InBody 770 analyzer. The device uses an eight-point tactile electrode system to measure 30 impedance values across five body segments (right and left upper limbs, trunk, and right and left lower limbs) at six frequencies (1 kHz, 5 kHz, 50 kHz, 250 kHz, 500 kHz, and 1,000 kHz). All measurements were scheduled between 09:00 and 12:00 each morning. To ensure measurement accuracy, the room temperature was maintained at 20 °C to 25 °C. Participants were instructed to avoid food intake and strenuous physical activity within 2 h before assessment, and to empty their bladder and bowel before measurement. During the assessment, participants were barefoot and wore light clothing. Metal accessories such as watches and keys were not permitted. Participants cleaned their palms and soles using electrolyte wet wipes as required by the device, then stood on the analyzer platform and held the hand electrodes, with the thumb and the other four fingers in firm contact with the electrodes. Both heels and forefeet were placed on the foot electrodes. Participants kept their arms relaxed and slightly abducted from the trunk, maintaining an angle of approximately 15 degrees between the upper limbs and the trunk, and remained standing in a relaxed posture. After entering participant identifiers and basic characteristics (participant number, name, sex, age, and height), the measurement was initiated and typically required 1 to 2 min. Participants were instructed to remain quiet and minimize movement during the assessment until all parameters were displayed and an audible signal indicated completion. The instrument automatically printed the measurement report, including body weight, BMI, body fat mass, total body water, protein mass, mineral content, visceral fat area, skeletal muscle mass, and bone mineral content.

### 2.3 Statistical analysis

All statistical analyses were performed using SPSS version 26.0 and R software. Continuous variables were assessed for distributional characteristics before analysis. Variables with an approximately normal distribution are presented as mean (SD), whereas skewed variables are presented as median (interquartile range), as appropriate. Categorical variables are presented as n (%). Between-group comparisons were performed using the independent-samples t test, Mann–Whitney U test, chi-square test, or Fisher’s exact test, as appropriate.

The distribution of FIB-4 was examined using histograms, Q-Q plots, skewness, and the Shapiro–Wilk test. To evaluate the suitability of linear regression models, residual diagnostics were performed for models using raw FIB-4 and log-transformed FIB-4 as outcomes. Model residuals were assessed using residual histograms, Q-Q plots, skewness, and the Shapiro–Wilk test. The dependent variable used in subsequent linear regression analyses was determined based on the distribution of raw FIB-4 and the residual diagnostics of models using raw and log-transformed FIB-4.

We used hierarchical multivariable linear regression models to evaluate the association of interest. Model 1 was unadjusted. Model 2 was adjusted for total cholesterol (TC), triglycerides (TG), and high-density lipoprotein cholesterol (HDL-C). Model 3 further adjusted for smoking history, history of hypertension, history of diabetes, and sex. Model 4 additionally adjusted for BMI. To further examine the role of BMI in this association, we conducted a bootstrap-based mediation analysis with 5,000 resamples. We estimated the total effect (path c), the direct effect (path c′), and the indirect effect through BMI (path a × b). BMI was defined as the mediator in the model. The statistical significance of the indirect effect was determined using the bootstrap 95% confidence interval, with an interval excluding zero considered statistically significant. A suppression effect was considered when the direct and indirect effects operated in opposite directions and the absolute value of the direct effect was greater than that of the total effect. All mediation effect estimates were reported on the same scale. All tests were two sided, and *p* < 0.05 was considered statistically significant.

## 3 Results

### 3.1 Baseline characteristics of the fatty liver disease cohort

A total of 661 participants were included, of whom 391 were men (59.15%). Participants aged 46 to 59 years accounted for 43.57% of the cohort. Baseline characteristics by sex are summarized in Table 1.

**Table 1:** Baseline characteristics of the study participants.

Variables	Overall (*N* = 661)	Female (*n* = 270)	Male (*n* = 391)	t/χ^2^	*p*
Age (year)	53.48 ± 11.22	56.97 ± 10.01	51.06 ± 11.38	7.043	<0.001**
Body composition parameters					
BMI (kg/m^2^)	26.50 ± 3.34	25.81 ± 3.38	26.98 ± 3.22	−4.480	<0.001**
PBF (%)	31.55 ± 6.47	36.44 ± 5.03	28.17 ± 5.04	20.753	<0.001**
WHR	0.95 ± 0.06	0.94 ± 0.06	0.96 ± 0.06	−3.017	0.003**
ASM (kg)	21.25 ± 4.76	16.90 ± 2.48	24.25 ± 3.46	−31.827	<0.001**
SMI (kg/m^2^)	7.5 ± 1.02	6.59 ± 0.68	8.12 ± 0.69	−28.292	<0.001**
SVR (kg/cm^2^)	0.21 ± 0.08	0.15 ± 0.05	0.25 ± 0.07	−21.451	<0.001**
Laboratory parameters					
PLT (10^^9^/L)	235.44 ± 56.06	247.36 ± 59.63	227.22 ± 51.96	4.610	<0.001**
ALT (U/L)	29.83 ± 20.95	26.38 ± 20.23	32.21 ± 21.13	−3.550	<0.001**
ALB (U/L)	44.51 ± 2.39	43.90 ± 2.24	44.93 ± 2.39	−5.584	<0.001**
AST (U/L)	21.70 ± 10.21	21.53 ± 10.15	21.82 ± 10.26	−0.350	0.727
TC (mmol/L)	5.10 ± 1.13	5.30 ± 1.07	4.97 ± 1.15	3.657	<0.001**
TG (mmol/L)	2.08 ± 2.21	1.90 ± 1.73	2.21 ± 2.48	−1.737	0.083
HDL-C (mmol/L)	1.18 ± 0.26	1.29 ± 0.26	1.11 ± 0.22	9.155	<0.001**
History of smoking, *n* (%)	91 (13.77)	2 (0.74)	89 (22.76)	65.240	<0.001**
History of hypertension, *n* (%)	248 (37.52)	111 (41.11)	137 (35.04)	2.513	0.113
History of diabetes, *n* (%)	98 (14.83)	39 (14.44)	59 (15.09)	0.053	0.819
FIB-4 Index	1.01 ± 0.48	1.09 ± 0.51	0.96 ± 0.45	3.448	0.001**
Low risk (FIB-4 < 1.3), *n* (%)	514 (77.76)	198 (73.33)	316 (80.82)	—	0.043
Intermediate risk (1.3 ≤ FIB-4 ≤ 2.67), *n* (%)	141 (21.33)	68 (25.19)	73 (18.67)		
High risk (FIB-4>2.67), *n* (%)	6 (0.91)	4 (1.48)	2 (0.51)		

*p < 0.05, **p < 0.01; Fisher’s exact test was used for FIB-4 risk categories.

Men had a higher mean BMI than women (26.98 ± 3.22 kg/m^2^ vs. 25.81 ± 3.38 kg/m^2^, *p* < 0.01), whereas women had a higher mean percentage body fat (36.44 ± 5.03% vs. 28.17 ± 5.04%, *p* < 0.01). Skeletal muscle-related measures were higher in men than in women, including ASM (24.25 ± 3.46 kg), SMI (8.12 ± 0.69 kg/m^2^), and SVR (0.25 ± 0.07 kg/cm^2^) (all *p* < 0.01). PLT, ALT, and ALB differed by sex, whereas AST did not. Women had higher PLT and lower ALT and ALB than men (all *p* < 0.001). Women also had higher TC and HDL-C than men (both *p* < 0.01), while TG did not differ significantly. Smoking prevalence was higher in men than in women (*p* < 0.01), whereas the prevalence of chronic comorbidities did not differ between sexes.

### 3.2 Differences in clinical characteristics by liver fibrosis risk category

**Figure 1:**
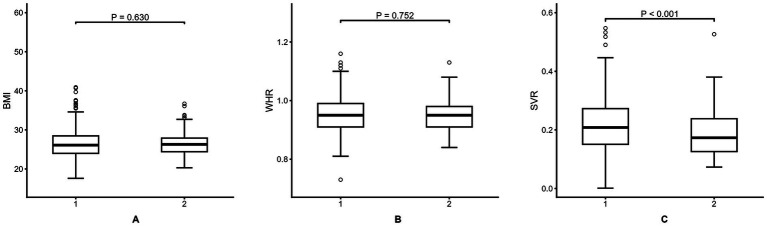
Box plots of body composition indicators by liver fibrosis risk category. 1 indicates the low-risk group, defined as FIB-4 < 1.3; 2 indicates the intermediate-to-high-risk group, defined as FIB-4 ≥ 1.3. *p* values were calculated using independent-samples t tests and are shown above the corresponding comparisons. BMI, body mass index; SVR, skeletal muscle-to-visceral fat area ratio; WHR, waist-to-hip ratio. **(A)** BMI; **(B)** WHR; **(C)** SVR.

Participants were categorized according to the FIB-4 thresholds into a low-risk group for liver fibrosis (*n* = 514) and an intermediate-to-high-risk group (*n* = 147) (Table 2). Statistically significant between-group differences were observed in demographic characteristics, body composition measures, and lipid parameters. Compared with the low-risk group, participants in the intermediate-to-high-risk group were older on average (*p* < 0.001) and had a higher proportion of women (*p* = 0.023). The prevalence of diabetes was higher in the intermediate-to-high-risk group, whereas the prevalence of smoking history was lower in this group. The prevalence of hypertension was numerically higher in the intermediate-to-high-risk group, although the difference was not statistically significant (Figure 1), mean BMI and WHR were similar between groups and did not differ significantly, whereas SVR was markedly lower in the intermediate-to-high-risk group than in the low-risk group (*p* < 0.001). Regarding lipid parameters, total cholesterol was lower in the intermediate-to-high-risk group than in the low-risk group (*p* < 0.001), while triglycerides and HDL-cholesterol did not differ significantly between groups (Table 2).

**Table 2:** Differences in clinical characteristics by liver fibrosis risk category.

Variables	FIB-4 risk category		*t*/χ²	*p*
	Low risk (*n* = 514)	Intermediate to high risk (*n* = 147)		
Age(year)	50.39 ± 10.04	64.27 ± 7.99	−17.495	<0.001**
Female, *n* (%)	198 (38.52)	72 (48.98)	5.174	0.023*
History of smoking, *n* (%)	80 (15.56)	11 (7.48)	6.288	0.012*
History of diabetes, *n* (%)	65 (12.65)	33 (22.45)	8.699	0.003*
History of hypertension, *n* (%)	184 (35.80)	64 (43.54)	2.921	0.087
Body composition parameters				
SVR (kg/cm^2^)	0.22 ± 0.08	0.19 ± 0.07	3.836	<0.001**
BMI (kg/m^2^)	26.53 ± 3.44	26.38 ± 2.95	0.482	0.630
WHR	0.95 ± 0.06	0.95 ± 0.06	0.316	0.752
Lipid parameters				
TC (mmol/L)	5.20 ± 1.13	4.78 ± 1.09	3.997	<0.001**
TG (mmol/L)	2.16 ± 2.34	1.82 ± 1.63	1.661	0.097
HDL-C (mmol/L)	1.18 ± 0.25	1.20 ± 0.27	−0.913	0.362

*p < 0.05, **p < 0.01. Values are presented as mean ± SD or n (%), as appropriate.

### 3.3 Association between SVR and liver fibrosis risk score

**Figure 2:**
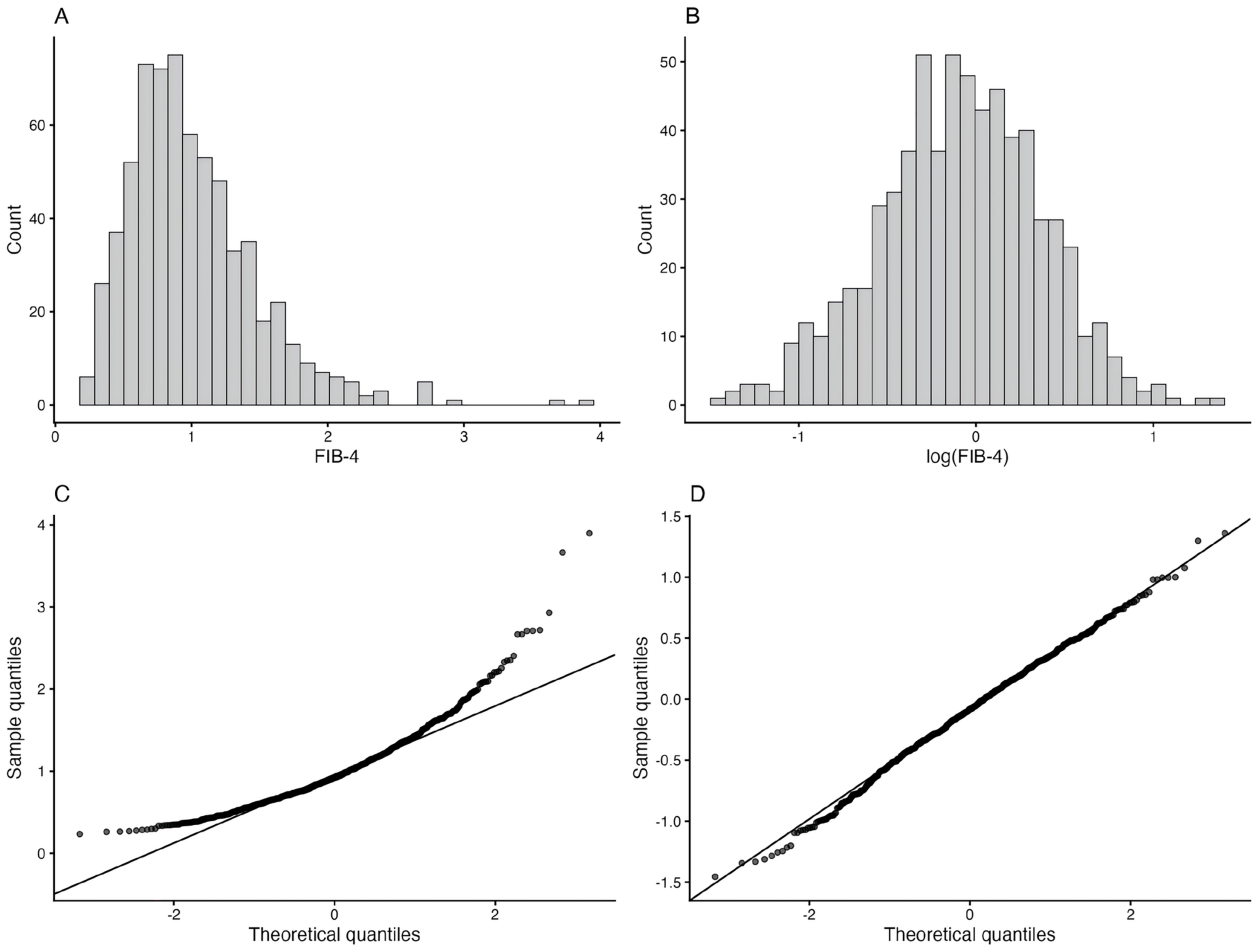
Distributional assessment of raw FIB-4 and log(FIB-4). Histograms and Q-Q plots showed that the raw FIB-4 score was markedly right-skewed and deviated from normality, whereas log(FIB-4) showed an approximately normal distribution after natural logarithmic transformation. FIB-4, the fibrosis-4 index. **(A)** Distribution of raw FIB-4; **(B)** Distribution of log(FIB-4); **(C)** Q-Q plot of raw FIB-4; **(D)** Q-Q plot of log(FIB-4).

Before linear regression analysis, the distribution of FIB-4 and model residuals was examined (Figure 2). The raw FIB-4 score was markedly right-skewed and deviated from normality (skewness = 1.440, Shapiro–Wilk *p* < 0.001). After natural logarithmic transformation, the distribution of log(FIB-4) was substantially improved and no longer showed significant departure from normality (skewness = −0.123, Shapiro–Wilk *p* = 0.444). Residual diagnostics further supported this transformation: residuals from the raw FIB-4 model were non-normally distributed (Shapiro–Wilk p < 0.001), whereas residuals from the log(FIB-4) model were approximately normal (Shapiro–Wilk *p* = 0.850). Accordingly, log(FIB-4) was used as the continuous outcome in subsequent linear regression analyses.

In the unadjusted model, SVR was inversely associated with log(FIB-4) (B = -0.067, 95% CI: −0.109 to −0.025, *p* = 0.002). After adjustment for lipid parameters, the association remained significant (B = −0.052, 95% CI: −0.095 to −0.010, *p* = 0.015). After further adjustment for smoking history, hypertension, diabetes, and sex, the association was attenuated and no longer significant (B = −0.017, 95% CI:−0.070 to 0.037, *p* = 0.537). When BMI was additionally included, the inverse association became significant again (B = −0.103, 95% CI: −0.167 to −0.040, *p* = 0.001) (Table 3).

**Table 3:** Hierarchical linear regression analysis of factors associated with the log(FIB-4).

Model	Variable	*β*	Standardized β	*t*	*p*	95% CI	Collinearity diagnostics	
							VIF	Tolerance
Model 1	Intercept	—	—	0.960	0.337	−0.049 ~ 0.141	—	—
	SVR	−0.067	−0.120	−3.108	0.002**	−0.109 ~ −0.025	NA	NA
Model 2	Intercept	—	—	−0.787	0.432	−0.332 ~ 0.142	—	—
	SVR	−0.052	−0.094	−2.427	0.015*	−0.095 ~ −0.010	1.041	0.960
	TC	−0.082	−0.201	−4.401	<0.001**	−0.118 ~ −0.045	1.455	0.687
	TG	0.023	0.110	2.342	0.019*	0.004 ~ 0.042	1.552	0.644
	HDL-C	0.406	0.225	4.886	<0.001**	0.243 ~ 0.569	1.481	0.675
Model 3	Intercept	—	—	−1.392	0.165	−0.426 ~ 0.073	—	—
	SVR	−0.017	−0.030	−0.618	0.537	−0.070 ~ 0.037	1.699	0.588
	TC	−0.070	−0.172	−3.753	<0.001**	−0.106 ~ −0.033	1.500	0.667
	TG	0.019	0.089	1.904	0.057	−0.001 ~ 0.038	1.573	0.636
	HDL-C	0.359	0.199	4.206	<0.001**	0.192 ~ 0.527	1.607	0.622
	Smoking	−0.064	−0.048	−1.203	0.230	−0.168 ~ 0.040	1.135	0.881
	Hypertension	0.083	0.088	2.256	0.024*	0.011 ~ 0.155	1.075	0.930
	Diabetes	0.159	0.123	3.228	0.001**	0.062 ~ 0.255	1.032	0.969
	Gender	−0.058	−0.062	−1.201	0.230	−0.152 ~ 0.037	1.893	0.528
Model 4	Intercept	—	—	3.289	0.001**	0.312 ~ 1.238	—	—
	SVR	−0.103	−0.185	−3.196	0.001**	−0.167 ~ −0.040	2.483	0.403
	TC	−0.066	−0.163	−3.615	<0.001**	−0.102 ~ −0.030	1.503	0.665
	TG	0.019	0.093	2.018	0.044*	0.001 ~ 0.038	1.574	0.635
	HDL-C	0.311	0.172	3.670	<0.001**	0.144 ~ 0.477	1.630	0.613
	Smoking	−0.058	−0.043	−1.109	0.268	−0.161 ~ 0.045	1.135	0.881
	Hypertension	0.102	0.108	2.803	0.005**	0.031 ~ 0.174	1.088	0.919
	Diabetes	0.154	0.119	3.194	0.001**	0.060 ~ 0.249	1.033	0.968
	Gender	0.060	0.064	1.117	0.264	−0.045 ~ 0.164	2.406	0.416
	BMI	−0.031	−0.222	−4.766	<0.001**	−0.043 ~ −0.018	1.602	0.624

*p < 0.05, **p < 0.01.

### 3.4 Mediation analysis of BMI in the association between SVR and log(FIB-4)

**Figure 3:**
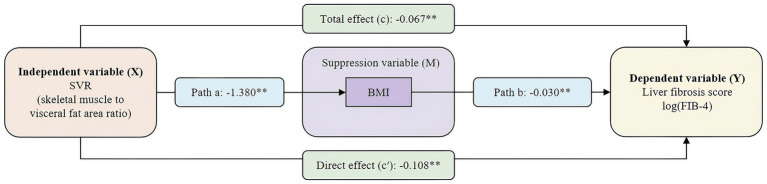
Mediation model of BMI in the association between SVR and log(FIB-4). BMI was defined as the mediator in the model. Path c indicates the total effect of SVR on log(FIB-4); path c′ indicates the direct effect of SVR on log(FIB-4) after accounting for BMI; path a × b indicates the indirect effect through BMI. The suppression effect refers to the observed mediation pattern in which the direct and indirect effects operated in opposite directions. *p < 0.05 **p < 0.01, ***p < 0.001. BMI, body mass index; FIB-4, fibrosis-4 index; SVR, skeletal muscle-to-visceral fat area ratio.

Given that inclusion of BMI substantially altered the estimated effect of SVR, we conducted a bootstrap-based mediation analysis with BMI defined as the mediator to clarify the role of BMI in this association. As shown in Figure 3 and Table 4, SVR had a significant direct inverse effect on log(FIB-4) (c′ = −0.108, bootstrap 95% CI: −0.149 to −0.067, *p* < 0.001). By contrast, the indirect effect through BMI was positive (a × b = 0.041, bootstrap 95%CI: 0.023 to 0.059, *p* < 0.001). The total effect was −0.067 (bootstrap 95% CI: −0.107 to −0.028, *p* = 0.001). Because the direct and indirect effects had opposite signs and the absolute value of the direct effect (0.108) exceeded that of the total effect (0.067), the pattern was consistent with a suppression effect. These findings suggest that BMI may suppress part of the inverse association between SVR and log(FIB-4), indicating that BMI should be considered when evaluating the implications of body composition for FIB-4-based risk assessment.

**Table 4:** Decomposition of total, direct, and indirect effects of SVR on log(FIB-4) with BMI as the mediator.

Item	Symbol	Definition	Effect estimate	Bootstrap 95% CI		*SE*	*p*
				Lower	Upper		
SVR= > BMI= > log(FIB-4)	a*b	Indirect effect	0.041	0.023	0.059	0.009	<0.001**
SVR= > log(FIB-4) (adjusted for BMI)	c’	Direct effect	−0.108	−0.149	−0.067	0.021	<0.001**
SVR= > log(FIB-4)	c	Total effect	−0.067	−0.107	−0.028	0.020	0.001**

All coefficients are unstandardized estimates. The indirect effect was tested using 5,000 bootstrap resamples. A bootstrap 95% confidence interval that did not include zero was considered statistically significant. **p < 0.01.

### 3.5 Subgroup mediation analyses of BMI in the association between SVR and log(FIB-4)

To further assess heterogeneity in the suppression effect of BMI, we conducted subgroup mediation analyses stratified by BMI level, sex, and the presence of common chronic conditions (Table 5). In the higher BMI subgroup (*n* = 508), we observed a significant suppression effect that was consistent with the overall findings. In the lower BMI subgroup (*n* = 153), however, the pattern did not meet criteria for a typical suppression effect. These results suggest that the suppressive role of BMI was primarily evident among participants with higher BMI. In sex-stratified analyses, both men (*n* = 391) and women (*n* = 270) showed a significant direct effect accompanied by a positive indirect effect via BMI, consistent with a suppression effect. When stratified by chronic disease status, a significant suppression effect was observed in both subgroups, with a slightly larger indirect effect in participants without chronic conditions (0.058) than in those with chronic conditions (0.053).

**Table 5:** Subgroup mediation analyses of BMI in the association between SVR and log(FIB-4).

Subgroup	Total (n, %)	Total effect (c) (95% CI)	Direct effect (c’) (95% CI)	Indirect effect (a*b) (95% CI)	Suppression effect
Overall	661	−0.067 (−0.107, −0.028)	−0.108 (−0.149, −0.067)	0.041 (0.023, 0.059)	Yes
BMI group					
Low BMI (<24 kg/m^2^)	153	−0.109 (−0.183, −0.044)	−0.117 (−0.191, −0.053)	0.008 (−0.003, 0.032)	Not significant
High BMI (≥24 kg/m^2^)	508	−0.060 (−0.109, −0.011)	−0.107 (−0.158, −0.057)	0.048 (0.026, 0.073)	Yes
Gender					
Male	391	−0.016 (−0.082, 0.047)	−0.102 (−0.185, −0.026)	0.087 (0.033, 0.145)	Yes
Female	270	−0.077 (−0.197, 0.018)	−0.201 (−0.362, −0.082)	0.124 (0.055, 0.208)	Yes
Presence of hypertension or diabetes					
Yes	297	−0.053 (−0.120, 0.007)	−0.091 (−0.157, −0.028)	0.037 (0.013, 0.067)	Yes
No	364	−0.058 (−0.108, −0.006)	−0.104 (−0.154, −0.053)	0.046 (0.025, 0.070)	Yes

Suppression effect was defined as opposite directions of the direct and indirect effects, with a statistically significant indirect effect based on the bootstrap 95% confidence interval.

## 4 Discussion

In this cross-sectional analysis of 661 individuals with fatty liver disease, higher SVR was associated with lower log(FIB-4), and this association became more apparent after accounting for BMI. The mediation analysis suggested an exploratory suppression pattern, in which BMI partially offset the inverse association between SVR and log(FIB-4). These findings should be interpreted as associations with a FIB-4-based non-invasive risk indicator rather than as direct evidence of histological fibrosis severity or causal hepatoprotection. Consistent with previous evidence (28), the protective association of SVR with FIB-4 was evident in the unadjusted model (total effect c = −0.067). After adjustment for smoking and chronic conditions, the association was attenuated. However, when BMI was additionally included, the direct protective association of SVR became more pronounced (c′ = −0.108). Mediation analysis further showed a positive indirect pathway through BMI (a × b = 0.041), which partially offset the direct benefit of SVR, resulting in a statistically defined suppression effect.

The association between SVR and FIB-4 should not be interpreted as a simple linear inverse relationship. Instead, our findings support a two-pathway framework in which the protective association of SVR is partly suppressed by BMI. The direct pathway (c′) suggests that a higher SVR, reflecting greater skeletal muscle mass and lower visceral adiposity, may confer hepatoprotection through improvements in insulin resistance, attenuation of systemic inflammation, and reduction of lipotoxicity (29). This interpretation is consistent with accumulating evidence linking low skeletal muscle mass to adverse liver-related outcomes (30, 31). By contrast, the indirect pathway through BMI (a × b) indicated that higher SVR tended to be associated with lower BMI, while lower BMI was associated with higher FIB-4 in our cohort. This pattern does not negate the established harms of obesity. Rather, it highlights that in individuals with lean fatty liver disease, clinical attention should focus on sarcopenia and pathological leanness, which may coexist with metabolic dysfunction and increase fibrosis risk (32). Without accounting for BMI, the clinical value of body composition measures could be substantially underestimated. Our subgroup analyses further suggested that the suppression effect of BMI was not evident in the lower BMI subgroup. The suppressive influence of BMI appeared strongest among participants with overweight or obesity, whereas in those with normal weight or underweight, the association between body composition and fibrosis risk might be more direct or driven by other factors. The suppression pattern was observed in both men and women, with a larger direct effect estimate in women. Similarly, the suppression effect was present regardless of the presence of common chronic conditions, indicating that this mechanism was robust across different metabolic backgrounds. These findings align with prior reports, including evidence that reduced skeletal muscle mass remains associated with adverse liver outcomes even after accounting for glycaemic status (18).

From a biological perspective, the observed suppression effect likely reflects the limitations of BMI as a coarse summary metric. BMI (weight divided by height squared) does not distinguish lean mass from fat mass and cannot differentiate subcutaneous from visceral adiposity. By contrast, a higher SVR captures a combination of greater skeletal muscle mass and lower visceral fat, which can be viewed as a favorable body composition profile with strong metabolic relevance. Skeletal muscle is a primary target tissue for insulin action and a major site of energy utilization. Higher muscle mass is generally associated with improved glucose and lipid handling, reduced insulin resistance, and lower metabolic burden on the liver (16). Visceral adipose tissue, in contrast, functions as an active endocrine organ that releases pro-inflammatory and pro-fibrotic mediators, thereby directly promoting hepatic inflammation and fibrogenesis (17). In our study, the inverse indirect pathway in which lower BMI was associated with higher FIB-4 is plausibly linked to phenotypes increasingly recognized among individuals with fatty liver disease, including sarcopenic obesity and malnutrition-related leanness. During the progression of chronic liver disease, protein–energy wasting and heightened muscle catabolism are common complications. Therefore, a lower BMI that primarily reflects loss of skeletal muscle, rather than reduction in adipose tissue, should not be interpreted as metabolic health. Instead, it may indicate more advanced disease and poorer prognosis. Our findings provide indirect support for this explanation, suggesting that the BMI reduction observed with higher SVR could attenuate the apparent hepatoprotective association if it is accompanied by clinically meaningful muscle loss.

In the clinical assessment and management of fatty liver disease, reliance on BMI alone is unlikely to capture the heterogeneity of metabolic risk. Body composition assessment methods that can distinguish skeletal muscle from adipose tissue, and visceral from subcutaneous fat, such as bioelectrical impedance analysis or computed tomography (CT) and magnetic resonance imaging (MRI), may enable more accurate risk stratification and earlier identification of NAFLD patients at risk of progressive liver fibrosis (8, 33). Previous validation studies comparing BIA-derived visceral fat area with CT-based measurements have reported acceptable correlations, although the absolute values obtained from the two methods may not be fully interchangeable (34, 35). From a clinical translation perspective, SVR may provide additional information beyond BMI in health examination settings. Because SVR integrates skeletal muscle mass and visceral adiposity, it may help identify individuals with fatty liver disease who have an unfavorable body composition profile despite similar BMI levels (14, 15, 20). When combined with FIB-4, SVR may support preliminary fibrosis risk stratification and help identify individuals who may benefit from further assessment, such as transient elastography, serum fibrosis markers, or specialist evaluation (24, 25).

However, because the present study was cross-sectional and observational, these findings should not be interpreted as direct evidence that modifying SVR can reduce liver fibrosis progression. Rather, they suggest that future longitudinal and interventional studies should examine whether improving body composition, particularly by reducing visceral adiposity and preserving skeletal muscle mass, can contribute to better liver-related outcomes. For patients with NAFLD who have normal or low BMI, future studies may further examine the value of sarcopenia screening and body composition-oriented interventions, such as nutritional support and resistance training, in fibrosis risk management (32).

Several limitations should be considered. First, this was a cross-sectional study, which precludes causal inference regarding the relationships among SVR, BMI, and liver fibrosis. Future work should validate these findings using longitudinal data. Second, although FIB-4 is widely used for non-invasive fibrosis risk stratification, it is less accurate than liver biopsy, strongly influenced by age, and should not be interpreted as a direct continuous measure of histological fibrosis severity. Because age is mathematically embedded in the FIB-4 formula, we did not include age as a separate covariate in the primary models to avoid adjusting for a component of the outcome itself. Therefore, the observed association between SVR and log(FIB-4) should not be interpreted as age-independent evidence of fibrosis risk. Although log transformation improved the distribution of FIB-4 and model residuals, the findings remain associations with a FIB-4-based non-invasive risk indicator (24). Third, although MASLD has become the updated terminology for fatty liver disease in recent consensus statements and guidelines (36), this study retained NAFLD as the operational diagnostic term because the original health examination records and participant identification were based on the NAFLD diagnostic framework. Given the substantial overlap between NAFLD and MASLD populations reported in previous studies, the present findings remain clinically interpretable; however, we did not fully reclassify participants according to the latest MASLD criteria. Therefore, future studies should validate these findings using MASLD-based definitions and more complete metabolic risk information. Fourth, participants were not uniformly screened for HBsAg or anti-HCV as part of the study protocol. Although individuals with documented or self-reported viral hepatitis were excluded, undiagnosed viral hepatitis could not be completely ruled out. This may have introduced potential misclassification and residual confounding. Fifth, although we adjusted for key confounders, residual confounding by factors such as diet, physical activity, and genetic predisposition cannot be excluded. Lastly, the subgroup analyses were based on relatively small sample sizes, particularly in the lower BMI subgroup (n = 153), which may have limited statistical power and reduced the stability of some estimates. Future studies should use prospective longitudinal designs and more precise assessments of liver fibrosis. Interventional studies are also warranted to determine whether improving SVR, through increasing skeletal muscle mass and reducing visceral adiposity, can directly slow liver disease progression.

## 5 Conclusion

Among individuals with fatty liver disease, higher SVR was associated with lower log(FIB-4), and BMI appeared to suppress part of this association in exploratory mediation analysis. These findings suggest that SVR may provide body-composition information beyond BMI in the assessment of FIB-4-based risk. The suppression effect was consistently observed in the higher BMI subgroup and in both men and women, but was not evident in the lower BMI subgroup, highlighting population heterogeneity in the relationship between body composition and liver health. These findings support the potential value of incorporating body composition assessment into fibrosis risk evaluation in fatty liver disease. However, longitudinal and interventional studies are needed to determine whether improving SVR can reduce liver fibrosis progression and contribute to better liver-related outcomes.

## Data Availability

The datasets generated and/or analyzed during the current study are not publicly available due to privacy and ethical restrictions but are available from the corresponding author on reasonable request and with appropriate institutional approval.

## Glossary

ALB: Albumin
ALT: Alanine aminotransferase
ASM: Appendicular skeletal muscle mass
AST: Aspartate aminotransferase
BMI: Body mass index
CVD: Cardiovascular disease.
CI: Confidence interval
CT: Computed tomography
FIB-4: Fibrosis-4 index
HCC: Hepatocellular carcinoma
HDL-C: High-density lipoprotein cholesterol
HC: Hip circumference
MRI: Magnetic resonance imaging
MASLD: Metabolic dysfunction-associated steatotic liver disease
NAFLD: Non-alcoholic fatty liver disease
NASH: Non-alcoholic steatohepatitis
PBF: Percentage body fat
PLT: Platelet count
SO: Sarcopenic obesity
SMI: Skeletal muscle index
SVR: Skeletal muscle-to-visceral fat area ratio
SD: Standard deviation
SE: Standard error
SPSS: Statistical Package for the Social Sciences
TC: Total cholesterol
TG: Triglycerides
VIF: Variance inflation factor
WC: Waist circumference
WHR: Waist-to-hip ratio
