# Supplementary material for: Association between the skeletal muscle-to-visceral fat area ratio and liver fibrosis risk in patients with non-alcoholic fatty liver disease: a cross-sectional study
Source: Front Public Health. 2026 Jul 15;14:1816286. doi: 10.3389/fpubh.2026.1816286 (PMC13415364; doi:10.3389/fpubh.2026.1816286)
Supplement: Supplementary file 1 [file Table_1.DOCX]

**Table S1. Age-adjusted sensitivity analysis for the association between SVR and log(FIB-4)**

| **Model** | **Adjustment** | **N** | **B (95% CI)** | **Standardized *β*** | ***p*** | **VIF** | **Tolerance** | ***R²*** | **Adjusted *R²*** | **AIC** | **BIC** | **Residual normality p** |
| --- | --- | --- | --- | --- | --- | --- | --- | --- | --- | --- | --- | --- |
| Primary adjusted model without age | Adjusted for TC, TG, HDL-C, smoking history, hypertension, diabetes, sex, and BMI | 661 | -0.103  (-0.167, -0.040) | -0.185 | 0.001 | 2.483 | 0.403 | 0.1185 | 0.1063 | 786.880 | 836.311 | 0.850 |
| Sensitivity model additionally adjusted for age | Age was additionally included although age is a mathematical component of FIB-4 | 661 | 0.063  (0.017, 0.108) | 0.112 | 0.007 | 2.676 | 0.374 | 0.5764 | 0.5699 | 304.488 | 358.413 | 0.070 |

The dependent variable was log(FIB-4). SVR was analyzed per 0.1-unit increase. The primary adjusted model did not include age. The sensitivity model additionally included age, but this model should be interpreted cautiously because age is a mathematical component of FIB-4. All coefficients are unstandardized estimates unless otherwise specified.
